# The Complexity of Bariatric Patient’s Pharmacotherapy: Sildenafil Biopharmaceutics and Pharmacokinetics before vs. after Gastric Sleeve/Bypass

**DOI:** 10.3390/pharmaceutics15122795

**Published:** 2023-12-18

**Authors:** Daniel Porat, Oleg Dukhno, Sandra Cvijić, Arik Dahan

**Affiliations:** 1 Department of Clinical Pharmacology, School of Pharmacy, Faculty of Health Sciences, Ben-Gurion University of the Negev, P.O. Box 653, Beer-Sheva 8410501, Israel; poratdan@post.bgu.ac.il; 2 Department of Surgery B, Soroka University Medical Center, Beer-Sheva 8410101, Israel; dukhnoo@bgu.ac.il; 3 Department of Pharmaceutical Technology and Cosmetology, Faculty of Pharmacy, University of Belgrade, Vojvode Stepe 450, 11221 Belgrade, Serbia; sandra.cvijic@pharmacy.bg.ac.rs

**Keywords:** bariatric surgery, oral absorption, gastric pH, ex vivo solubility, postbariatric dissolution, delayed onset, phosphodiesterase-5 inhibitors, physiologically based pharmacokinetic modeling, erectile dysfunction

## Abstract

Postbariatric altered gastrointestinal (GI) anatomy/physiology may significantly harm oral drug absorption and overall bioavailability. In this work, sildenafil, the first phosphodiesterase-5 (PDE5) inhibitor, was investigated for impaired postbariatric solubility/dissolution and absorption; this research question is of particular relevance since erectile dysfunction (ED) is associated with higher body mass index (BMI). Sildenafil solubility was determined both in vitro and ex vivo, using pre- vs. postsurgery gastric contents aspirated from patients. Dissolution tests were done in conditions mimicking the stomach before surgery, after sleeve gastrectomy (post-SG, pH 5), and after one anastomosis gastric bypass (post-OAGB, pH 7). Finally, these data were included in physiologically based pharmacokinetic (PBPK) modelling (GastroPlus^®^) to simulate sildenafil PK before vs. after surgery. pH-dependent solubility was demonstrated with low solubility (0.3 mg/mL) at pH 7 vs. high solubility at pH 1–5, which was also confirmed ex vivo with much lower solubility values in postbariatric gastric samples. Hampered dissolution of all sildenafil doses was obtained under post-OAGB conditions compared with complete (100%) dissolution under both presurgery and post-SG conditions. PBPK simulations revealed delayed sildenafil absorption in postbariatric patients (increased t_max_) and reduced C_max_, especially in post-OAGB patients, relative to a presurgery state. Hence, the effect of bariatric surgery on sildenafil PK is unpredictable and may depend on the specific bariatric procedure. This mechanistically based analysis suggests a potentially undesirable delayed onset of action of sildenafil following gastric bypass surgery.

## 1. Introduction

Obesity is a major factor contributing to the development of serious medical conditions, including diabetes, hyperlipidemia, and cardiovascular disease. Obesity is also associated with other medical issues such as erectile dysfunction (ED). The prevalence of ED is higher in men with a body mass index (BMI) of 25–30 kg/m^2^, compared to men with a BMI below 25 kg/m^2^, and higher still in men with a BMI over 30 kg/m^2^ [1]. This association is related to both pathophysiological and psychological factors. Pathophysiological factors include reduced synthesis of nitric oxide (NO), lower plasma testosterone levels, endothelial dysfunction, and dyslipidaemia [2]. Psychological factors include low self esteem, stress, and anxiety associated with excessive weight.

Bariatric surgery is a highly effective approach towards the treatment of patients with severe obesity. Significant and long-lasting weight loss is achieved in many cases, as well as the resolution of related comorbidities. As of 2023, there are several bariatric surgery procedures commonly performed, including sleeve gastrectomy (SG), one-anastomosis gastric bypass (OAGB), and Roux-en-Y gastric bypass (RYGB). Bariatric surgery was also shown to have a positive effect on sexual satisfaction and erectile function [3,4,5,6]. This may be related to weight loss [7,8] and overall improved body image [9,10]. However, in many cases, erectile dysfunction may still be a problem after these surgeries.

The first line and most common treatment of erectile dysfunction nowadays is oral phosphodiesterase-5 inhibitors (PDE5i), with attributes of high safety, rapid efficacy and noninvasiveness [11,12]. Following sexual stimulation, NO is released from endothelial cells. NO interacts with guanylyl cyclase in smooth muscle cells, resulting in the synthesis of cGMP from GMP. PDE5i prevents the hydrolysis of cGMP by PDE5, thus allowing penile smooth muscle relaxation, leading to increased arterial blood flow and erection [13]. However, to be effective, these drugs require satisfactory absorption from the gastrointestinal tract (GIT) into the bloodstream, a process dependent upon various factors related to the drug properties, the drug product (formulation, excipients), and the GIT physiology [14,15].

Following bariatric surgery, the GIT anatomy/physiology is significantly altered. 80–90% of the stomach is excised, resulting in decreased gastric volume, acidity, contractility, and residence time [16,17]. These changes may severely hamper the dissolution of the drug dose, a prerequisite for drug absorption [18,19]. Some drugs, including those with limited water solubility, high dose, and a weakly basic nature, are more prone and may be more sensitive to these postbariatric changes than others [20,21].

Sildenafil is the first and main PDE5i, approved for ED treatment, as well as for pulmonary arterial hypertension. It has a weakly basic, pH-dependent solubility nature [22]. Sildenafil is among the 200 most commonly prescribed medications in the US (2020) and the 1st among all ED drugs, with ~3 million prescriptions annually (ClinCalc.com (accesses on 15 December 2023); therefore, it is clinically relevant and significant to study the use of this drug in the growing population of bariatric patients (∼700,000 surgeries annually worldwide). The pH-dependent solubility of sildenafil was studied in vitro, as well as ex vivo, in aspirated gastric contents obtained from patients pre- vs. postbariatric surgery. Then, a recently developed biorelevant in vitro dissolution technique was used to mimic the post-SG/OAGB gastric conditions and study the sildenafil product (Viagra^®^) dissolution pre- vs. postbariatric surgery. Finally, advanced physiologically based pharmacokinetic (PBPK) models were developed to simulate the absorption and pharmacokinetic changes in sildenafil pre- vs. postbariatric surgery. Altogether, this mechanistic research reveals the complexity involved in pharmacotherapy management after bariatric surgery, attempting to predict potential postbariatric treatment failure in advance for the benefit of this growing patient population.

## 2. Materials and Methods

### 2.1. Materials

Sildenafil citrate powder (Carbosynth Limited, Berkshire, UK) was used for the solubility studies. Sildenafil citrate (Viagra^®^, Pfizer Inc., New York, NY, USA) tablets of 25, 50 and 100 mg were used in the dissolution tests. The following materials from Sigma-Aldrich (Chemie GmbH, Steinheim, Germany) were used for buffer preparation: acetic acid, maleic acid, sodium phosphate monobasic, sodium dodecyl sulfate (SDS), hydrochloric acid, sodium hydroxide, and sodium chloride. Water and acetonitrile of ultraperformance liquid chromatography (UPLC)-grade were purchased from Bio-Lab Ltd. (Ashkelon, Israel).

### 2.2. In Vitro Solubility

The equilibrium solubility of sildenafil was determined using the shake-flask method, described previously [23,24]. pH 1 and pH 3 (maleate 0.2 M), pH 5 (acetate 0.2 M), and pH 7 (phosphate 0.2 M) buffers were used. Quadruplets (*n* = 4) for each pH were produced by excess drug powder added to vials containing 500 µL of the solution medium. Vials were incubated for 24 h at 37 °C and shaken at 200 rpm (Orbital Shaker Incubator, MRC Laboratory Instruments, Holon, Israel). Next, the samples were then moved to Eppendorf tubes and centrifuged (Centrifuge 5430 R, Eppendorf^®^, Hamburg, Germany) at 20,817× *g* and 37 °C for 10 min. Then, supernatants were diluted as needed (by a factor of 10 or 40, depending on solubility) and immediately analyzed by UPLC-PDA.

### 2.3. Ex Vivo Solubility

The solubility of sildenafil in gastric fluid was determined using the above-mentioned shake-flask method. Excess drug powder was added to glass vials containing gastric fluid at a fasted state, aspirated from three bariatric patients (one SG, one RYGB, and one OAGB) in the perioperative time through the nasogastric tube, before and one day after surgery. The study protocol was approved by the institutional review board of the Ben-Gurion University School of Medicine (institutional board request number 0248-18-SOR) and informed consent was obtained presurgery from all patients. pH measurements and recording were done immediately after gastric content collection; the content was vortexed, and 2 mL of gastric fluid were centrifuged for 10 min at 20,817× *g* and 25 °C; 400 µL of supernatant fluid was used in the solubility experiment [20,21]. Samples with appropriate pH [16] and sufficient gastric fluid volume were chosen for the solubility study. The experimental process and conditions were similar to the solubility study in vitro (Section 2.2) with the additional step of sample filtering prior to UPLC analysis. Dilutions were made with an acidic buffer after the removal of any undissolved drug.

### 2.4. In Vitro Dissolution

The dissolution of sildenafil, 25, 50 and 100 mg whole tablets, was studied in three different conditions: (1) pH 1 maleate, 250 mL medium, using USP dissolution apparatus II (Premiere 5100, Distek^®^, North Brunswick, NJ, USA), and with a paddle rotated at 100 rpm [25,26]; (2) pH 5 acetate, 50 mL (+0.05% SDS) medium; and (3) pH 7 phosphate, 50 mL (+0.05% SDS) medium, using 50 mL round-bottom flask inside a water bath with a minipaddle rotated at 153 rpm. This rotation-speed calculation was previously described [20,21,27]. SDS was used to prevent precipitation of the dissolved drug. A temperature of 37 ± 0.5 °C was maintained throughout the dissolution study. These conditions mimic the intragastric parameters before and after bariatric surgery, including pH, fluid volume, temperature, and gastric contractility. Samples of 300 µL were drawn at 5, 10, 15, 20, 30, 40, 50, 60, 75, and 90 min, filtered, and centrifuged for 12 min at 20,817× *g* (37 °C) before UPLC analysis.

### 2.5. Analytical Method

Sample analysis was performed using UPLC with Waters Acquity H-Class system equipped with a PDA detector and controlled by Empower software (EMP 2 Feature release 5, Built 2154). The analytical method followed the previous publication [28], and it is detailed in Table 1. The ambient temperature of the column and samples was used. Linear (R^2^ = 0.999) calibration curves were obtained for each pH in the relevant drug-concentration ranges. Inter- and intraday coefficients of variation were lower than 1%. The stability of sildenafil over the experimental course was verified.

### 2.6. Physiologically Based Pharmacokinetic (PBPK) Modeling

Drug-specific PBPK models were constructed using GastroPlus^®^ software (version 9.8.3012; Simulations Plus Inc., Lancaster, CA, USA). The software predicts the dissolution, absorption, and disposition of a drug based on its physicochemical and PK properties, in conjunction with the parameters describing the physiological characteristics of the human gastrointestinal (GI) tract. These physiological parameters are integrated into the software-specific advanced compartmental absorption and transit (ACAT) model of the GI tract and accounted for in a series of differential equations used to simulate the dynamic processes a drug undergoes in the body after peroral administration [29,30]. Physiological parameters describing a healthy human representative in the fasted state were kept at the software default values, except for the % fluid volumes in the small intestine (23%) and colon (0.5%), which were decreased from the default 40% and 10% to 23% and 0.5% [31], respectively, to account for the much smaller GI volumes in vivo [32,33,34]. To account for postbariatric surgery changes in physiological conditions, the ACAT model parameters were manually adjusted, i.e., gastric volume was decreased from default 50 to 10 mL, corresponding to 20% of the presurgery gastric volume [35,36], and gastric transit time was decreased from default 0.25 to 0.12 h [37,38]. The simulations were also performed for bypassed duodenum and jejunum physiology in post-OAGB patients, setting the volume, length, and transit time for the duodenum and jejunum 1 and 2 segments to zero [20,36]. Moreover, stomach pH was increased from default pH 1.3 to pH 5.0 (post-SG) and pH 7.0 (post-OAGB) to account for the influence of the different bariatric procedures and gastric pH on drug dissolution and absorption [16,39]. Intestinal pH was not altered [39].

The input values regarding properties of sildenafil were taken from the literature, in silico predicted (ADMET Predictor^®^ module, version 10.4.0.0; Simulations Plus Inc., Lancaster, CA, USA) based on the structure of the drug or experimentally determined, as depicted in Table 2. Drug dissolution throughout the GI tract was calculated using the software default Johnson equation [40] that accounts for a drug’s solubility and particle size. In the absence of data on drug particle size in the commercial products, an approximate value of 100 µm was used for all simulations. Considering the poor aqueous solubility, the models also accounted for the effect of bile salts on drug solubility and dissolution.

Pharmacokinetic parameters describing the distribution and elimination of the tested drug were estimated from the in vivo data on drug plasma concentration over time following intravenous (i.v.) and/or peroral drug dosing, using the software integrated PKPlus^TM^ module and, if needed, further optimized, while keeping the final values within the range reported in literature Table 2.

The predictive power of the designed models was assessed by comparing the predicted with the in vivo observed values for the maximum plasma concentration (C_max_), time to reach C_max_ (t_max_) and area under the plasma concentration time curve (AUC_0−inf_) for different drug doses given to healthy human subjects in the fasted state. The predicted values referred to a healthy human representative, and the in vivo values were digitized (DigIt software, version 1.0.4; Simulations Plus, Inc., Lancaster, CA, USA) from the mean profiles observed in clinical studies and published in the literature [41,42,43]. A comparison was performed by calculating fold errors between the observed and predicted data, whereas the fold error represents the ratio between the predicted and observed values. According to the commonly applied criteria, a 2-fold error can be considered acceptable for most drugs, although tighter boundaries in the 1.5-fold range could be more appropriate for drugs with, e.g., low variable pharmacokinetics and vice versa, less stringent 2.5-fold criterium may apply for drugs with high pharmacokinetic variability [44,45]. Additionally, the coefficient of determination (R^2^) was used to evaluate linearity between the observed and predicted values.

**Table 2:** Input parameters for sildenafil PBPK models.

	Value	Source/Ref
**Molecular weight (g/mol)**	474.59	/
**LogP/LogD**	2.7 (pH 7.4)	[46]
**Solubility at 37 °C (mg/mL)**	30 (pH 1); 2 (pH 3); 3.1 (pH 5); 0.3 (pH 7)	Experimental
**pKa(s)**	1.72 (base); 6.03 (base); 8.74 (acid)	predicted using ADMET Predictor^®^ module, then fitted (software integrated option) to the experimental pH solubility profile
**Human effective permeability, Peff (cm/s)**	4.48 × 10^−4^	[47]
**Diffusion coefficient (cm^2^/s)**	0.6006 × 10^−5^	software calculated (based on drug molecular weight)
**Particle diameter (µm)**	100	Approximated
**Mean precipitation time (s)**	900	software default
**Drug dose (mg) (dosage form)**	20 ^a^, 25, 100 (tablet); 50 (tablet, capsule ^a^)	/
**Volume of fluid taken with drug (mL)**	250 (presurgery); 50 (postsurgery)	software default or decreased by 80% to comply with the smaller gastric volume [35] and limited volume of fluid the bariatric patient can ingest
**Blood/plasma concentration ratio**	0.81	predicted using ADMET Predictor^®^ module
**Plasma fraction unbound (%)**	4	[46,48]
**First pass effect, FPE (%)**	60	optimized to comply with the literature’s reported values [46,49,50]
**Clearance, CL (L/h/kg)**	0.556	estimated using PKPlus^TM^ module, based on the in vivo data for i.v. drug dose [42]; comply with the reported data [41]
**Volume of distribution, Vd (L/kg)**	1.112	
**Distribution constant k_12_ (1/h)**	0.049	
**Distribution constant k_21_ (1/h)**	0.292	
**Elimination half life, t_1/2_ (h)**	2.83	software calculated; complies with the reported data [46,49]

^a^ solely for the model validation.

## 3. Results

### 3.1. Sildenafil Solubility

**Figure 1:**
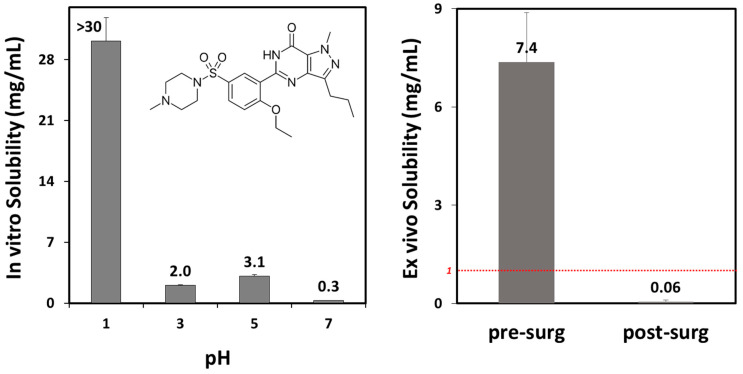
Sildenafil in vitro solubility as a function of pH ((**left**) panel) and ex vivo solubility in gastric fluid aspirated from three patients before ((**left**) column; mean pH 2.0) vs. after ((**right**) column; mean pH 7.0) bariatric surgery. The red dashed line represents the solubility threshold for complete postbariatric dose dissolution. Data presented as mean (SD); *n* = 4 for each pH; *n* = 3 for each experimental group.

The in vitro solubility of sildenafil decreased with the increasing pH. At pH 1, the solubility was over 30 mg/mL; however, at pH 7, sildenafil had low solubility, 0.3 mg/mL, representing an over 100-fold decrease. The ex vivo solubility in intragastric contents from pre- vs. postbariatric patients (Table 3) was consistent with the in vitro solubility results showing a >100-fold decreased solubility for sildenafil postsurgery (Figure 1; Table 4).

### 3.2. In Vitro Dissolution

**Figure 2:**
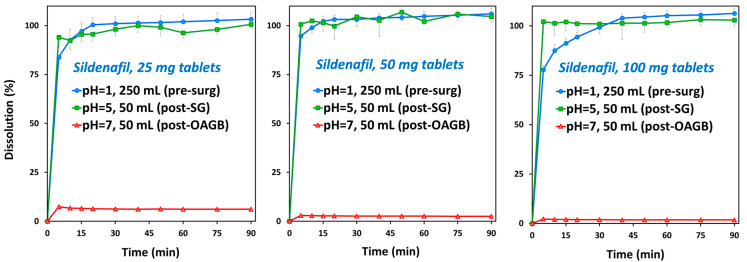
Sildenafil in vitro dissolution of commercially available Viagra^®^ tablets of 25 mg ((**left**) panel), 50 mg ((**middle**) panel) and 100 mg ((**right**) panel). The 250 mL at pH 1 medium (blue circles) represents presurgery stomach state, 50 mL at pH 5 medium (green squares) represents post-SG conditions, and 50 mL at pH 7 medium (red triangles) represents post-OAGB gastric scenario. Average (SD); *n* = 4 for each experimental group.

The dissolution of sildenafil was complete (100%) in presurgery, pH 1 conditions and in post-SG, pH 5 conditions. However, in post-OAGB, pH 7 conditions, sildenafil dissolution was severely hampered, with less than 10% of the sildenafil dissolved from its drug products (Figure 2). In other words, while in presurgery conditions, the dissolution of sildenafil is complete, in postsurgery conditions, sildenafil dissolution depends on the pH.

### 3.3. Physiologically Based Pharmacokinetic (PBPK) Simulations

**Figure 3:**
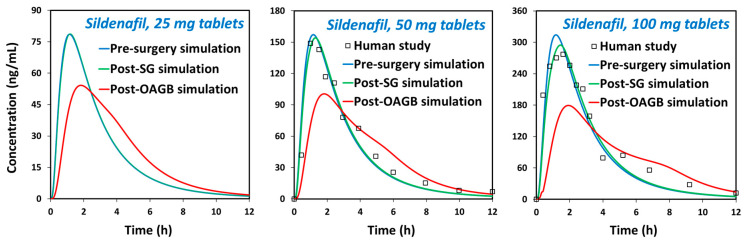
The predicted plasma concentration time profiles under presurgery, post-SG, and post-OAGB states for sildenafil 25 mg ((**left**) panel), 50 mg ((**middle**) panel), and 100 mg ((**right**) panel). Square markers represent the mean values observed in human studies (50 mg, Nichols et al.; 100 mg, Alwhaibi et al.) [41,49].

The simulated pharmacokinetic parameters (C_max_, t_max_, AUC_0−inf_) for different oral doses of sildenafil coincide well with the mean data observed in clinical studies (Supplementary Table S1), demonstrating the acceptable prediction power of the designed PBPK models. The presurgery simulated plasma concentrations relate well with the available data from human studies, both for 50 mg and 100 mg sildenafil doses (Figure 3). According to the calculated R^2^ values (Supplementary Table S1), there is a linear relationship between the simulated and observed data. The predictions for sildenafil are within the 1.5-fold range (0.67–1.50), except for AUC_0−inf_ for 100 mg sildenafil dose (fold error 0.66) which complies with the two-fold criterion (range between 0.5 and 2.0). A larger deviation of the predicted AUC_0−inf_ from the mean in vivo data in the case of 100 mg sildenafil dose can be explained by a slightly nonproportional increase in systemic drug exposure with an increased drug dose, which was attributed to the saturation of elimination pathways [51]. However, the degree of nonproportionality for doses up to 200 mg is considered small and not clinically significant [49,51]. Moreover, sildenafil exhibits pronounced interindividual pharmacokinetic variability [50] implying that a two-fold prediction error is acceptable for this compound.

The designed drug-specific PBPK models were eventually used to estimate in vivo dissolution and absorption of sildenafil in postbariatric vs. nonoperated subjects and to mechanistically explain the combined influence of drug physicochemical and physiological factors on its systemic exposure.

**Figure 4:**
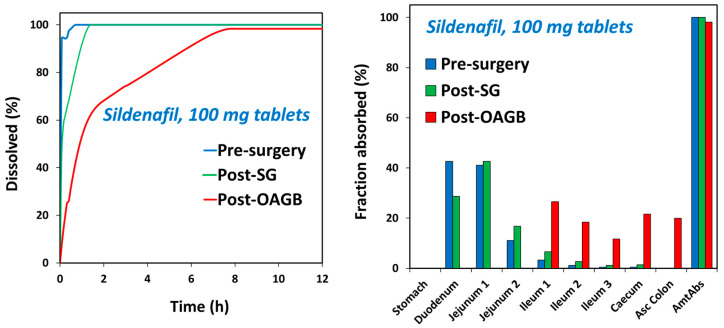
The predicted in vivo dissolution ((**left**) panel) and the predicted GI tract regional absorption ((**right**) panel) profiles under presurgery, post-SG, and post-OAGB states for sildenafil 100 mg tablets.

The prediction results for sildenafil (Figure 3, Supplementary Table S2) indicate delayed drug absorption in postbariatric patients (increased t_max_) and reduced C_max_ values, especially in post-OAGB patients in comparison to a presurgery state. Moreover, the reduction in C_max_ was more pronounced with an increasing drug dose, reaching a 43% reduction in a post-OAGB state for a 100 mg sildenafil dose. Another observation regarding sildenafil plasma exposure is that the increase in stomach pH from pH 5 to pH 7 reduces C_max_ and prolonges t_max_, which can be explained by delayed overall drug dissolution in cases of less acidic stomach pH (Figure 4, left panel). These results are in line with the experimental data showing a notable decline in drug solubility and postsurgery gastric dissolution when the pH changes from pH 5 to pH 7 (Figure 1 and Figure 2). As a consequence of reduced gastric dissolution, there is a marked drop in sildenafil absorption from the subsequent GI compartment (duodenum in post-SG patients and ileum 1 in post-OAGB patients), as visible from the simulated regional absorption distribution (Figure 4, right panel). However, the overall sildenafil plasma exposure (total percent absorbed in Figure 3 and AUC in Supplementary Table S2) does not seem to be affected by the altered GI conditions after bariatric surgery. Namely, according to the simulation results, even a 100 mg drug dose will eventually dissolve in the GI tract, and high drug permeability will enable sufficient drug absorption from the subsequent GI compartments (Figure 3 and Figure 4, right panel).

## 4. Discussion

This work shows that the solubility of sildenafil may be significantly lower in postbariatric conditions, resulting in potentially hampered dissolution of the drug dose. For sildenafil, the postoperative gastric pH is a crucial factor. This observation is attributed to its pKa value of ~6.7 [52], so that highly increased intragastric pH, such as that measured for some postbariatric patients (especially those undergoing bypass procedures) may result in dramatically lower solubility [16]. In fact, low solubility was observed in high pH (~7) gastric fluids from patients after different bariatric procedures. This indicates that, while some patients may have unaltered sildenafil pharmacokinetics after bariatric surgery, others may experience treatment failure related to insufficient drug absorption and exposure [20,53].

Bariatric surgery may prolong the absorption half life, especially in bypass procedures. For many orally administered drugs, absorption occurs from the upper part of the small intestine [54,55], and, when bypassed, the full drug dose may only be absorbed later, as distally as the large intestine [20,56]. It should be highlighted that, particularly with sildenafil, the onset of action is desired to be as immediate as possible, and, hence, altered/delayed absorption (with lower C_max_ and longer T_max_) following gastric bypass is detrimental to treatment success, even with an unaffected AUC.

Sildenafil is increasingly popular, and, in the US as of 2020, sildenafil had ~3 million prescriptions [57]. As mentioned, people with obesity are even more likely to experience ED and be treated with PDE5i. Bariatric surgery may improve sexual function among people with obesity; however, no data is available on changes in PDE5i use after surgery [58]. It is likely that many people will still require PDE5i after bariatric surgery. For one, weight loss along with its beneficial effects is not immediate, so continuation is expected for at least the first few months [59]. Second, when the underlying cause of ED is unrelated to weight, then the surgery itself is unlikely to solve the problem. Third, drugs taken by the patients may cause ED, e.g., in psychiatry; a high percentage of people undergoing bariatric surgery have a psychiatric background [60]. In fact, one year after bariatric surgery, the prevalence of psychiatric conditions is even higher than prior to surgery [61]. Many of the most commonly prescribed psychotropic drugs are likely to cause ED, so for these patients, erectile problems may well persist [62]. Fourth, while long-term weight loss is achieved in many cases, the phenomenon of weight regain is well known and quite common [63]. For all these reasons, PDE5i use, and in particular sildenafil, is expected to be highly relevant among patients who underwent bariatric surgery.

Previous studies predicted hampered dissolution, resulting in decreased blood levels for weakly basic drugs with particularly low pKa. These drugs were lamotrigine [21] (pKa = 5.7), loratadine [20] (pKa = 4.5), and etoricoxib (pKa = 4.6) [56]. Here, we showed that a basic drug with pKa in the upper limit of the physiological range (pKa ~6.7) is also sensitive to increased intragastric pH after bariatric surgery, suggesting that multiple other drugs with similar pKa values may also have altered dissolution and absorption following these procedures. One such drug is vardenafil, another important PDE5i; given the high structural similarity and common basic nature, the postbariatric predictions for sildenafil may apply to vardenafil, as well. In this context, no reported interactions between sildenafil/vardenafil and proton pump inhibitors (PPIs) can be found in the literature. This may be so, given that bariatric surgery also involved the removal of at least 80% of the stomach volume, further contributing to the limited solubility/dissolution. This is not the case in patients not having bariatric surgery who use PPIs.

Other mechanisms involved in the pharmacokinetics of these drugs may also be affected by bariatric surgery [64,65,66,67,68]. For example, the activity of CYP3A enzymes is increased with weight loss [69,70,71], and since sildenafil is mainly metabolized by CYP3A4, its postbariatric plasma levels may be further decreased.

It is worth mentioning that another drug in this class, udenafil, is currently available in Korea, Russia, and the Philippines and is yet to be approved for use in the USA by the FDA. It is structurally close to sildenafil and vardenafil but is a stronger base and also contains a weakly acidic group. Thus, it is expected to be far less affected by altered intragastric pH after bariatric surgery. It was also found to be weight negative in mice studies [72], implying suitability for this patient population.

## 5. Conclusions

Delayed absorption of sildenafil after gastric bypass surgery may suggest that ingestion of this drug shortly before intercourse, as normally indicated, may fail to produce the desired effects. In fact, after gastric bypass, patients using sildenafil may need to take this drug hours in advance, which is suboptimal and impractical, to say the least. Time to effect may change from patient to patient, so predicting the proper timing for taking it may not be possible. Indeed, when PDE5i is considered, the absorption rate is as important as its extent.

The issues addressed in this work should be further elucidated in human studies of the pharmacokinetics and pharmacodynamics of the different PDE5i among patients undergoing bariatric surgery, with special emphasis on the comparison between the different bariatric procedures, most specifically sleeve gastrectomy vs. gastric bypass.

## Figures and Tables

**Table 1:** UPLC-PDA analytic method for solubility/dissolution studies of sildenafil.

Drug	Column	Mobile Phase	Flow Rate (mL/min)	Injection Volume (µL)	Total Run Time (min)	Retention Time (min)	Detection Wavelength (nm)
Sildenafil	Waters XBridge C8, 3.5 µm, 4.6 × 150 mm	Water:Acetonitrile (+0.1% trifluoroacetic acid), 90:10 to 15:85 (*v*/*v*), gradient	1.0	5	5.0	4.0	294

**Table 3:** Patient characteristics include measured intragastric pH before vs. day after bariatric surgery. RYGB, Roux-en-Y gastric bypass; OAGB, one-anastomosis gastric bypass; SG, sleeve gastrectomy.

Patient	Age	Gender	BMI	Procedure	Presurg pH	Postsurg pH
1	49	male	43	RYGB	2.7	6.8
2	51	female	39	OAGB	1.5	7.2
3	25	male	71	SG	2.0	7.0

**Table 4:** In vitro and ex vivo saturation solubility of sildenafil. Data presented as mean (SD); in vitro, *n* = 4; ex vivo, *n* = 3.

		In Vitro				Ex Vivo	
**Conditions**	**Medium**	**Maleate Buffer**	**Maleate Buffer**	**Acetate Buffer**	**Phosphate Buffer**	**Presurgery Stomach Content**	**Postsurgery Stomach Content**
	**pH**	1	3	5	7	2.1 (0.6)	7.0 (0.2)
**Solubility** **(mg/mL)**		>30	2.0 (0.09)	3.1 (0.2)	0.3 (0.03)	7.4 (1.5)	0.06 (0.05)

## Data Availability

Data will be made available upon reasonable request.

## Supplementary Materials

The following supporting information can be downloaded at: https://www.mdpi.com/article/10.3390/pharmaceutics15122795/s1, Table S1: Comparison of the simulated and observed pharmacokinetic parameters for sildenafil; Table S2: Percent decrease/increasea in the simulated values of pharmacokinetic parameters for sildenafil before and after bariatric surgery in comparison to pre-surgery state.
