# Supplementary material for: The Complexity of Bariatric Patient’s Pharmacotherapy: Sildenafil Biopharmaceutics and Pharmacokinetics before vs. after Gastric Sleeve/Bypass
Source: Pharmaceutics. 2023 Dec 18;15(12):2795. doi: 10.3390/pharmaceutics15122795 (PMC10747454; doi:10.3390/pharmaceutics15122795)
Supplement: Supplementary file 1 [file pharmaceutics-15-02795-s001.zip › pharmaceutics-2709004-supplementary.pdf]

Table S1. Comparison of the simulated and observed pharmacokinetic parameters for sildenafil

20 mg	Observed[a]	Predicted	Fold error	R^2 value
C_{max} (ng/ml)	67.10	62.83	0.94	0.9637
t_{max} (h)	0.78	1.12	1.44	
AUC_{0-inf} (ng h/ml)	204.70	205.55	1.00	
**50 mg**	**Observed[a]**	**Predicted**	**Fold error**	
C_{max} (ng/ml)	147.00	162.16	1.10	
t_{max} (h)	1.01	1.01	1.00	
AUC_{0-inf} (ng h/ml)	519.10	513.87	0.99	
**100 mg**	**Observed[a]**	**Predicted**	**Fold error**	
C_{max} (ng/ml)	277.00	314.09	1.13	
t_{max} (h)	1.75	1.20	0.69	
AUC_{0-inf} (ng h/ml)	1559.30	1027.70	0.66	

[a] Estimated from the mean plasma concentration–time profiles

Table S2. Percent decrease/increase[a] in the simulated values of pharmacokinetic parameters for sildenafil before and after bariatric surgery in comparison to pre-surgery state

25 mg	Post-SG (gastric pH=5)	Post-OAGB (gastric pH=7)
C_{max}	-0.32	31.00
t_{max}	-7.14	-64.29
AUC_{0-inf}	0.00	0.28
**50 mg**	**Post-SG (gastric pH=5)**	**Post-OAGB (gastric pH=7)**
C_{max}	1.87	36.00
t_{max}	-14.29	-64.29
AUC_{0-inf}	0.00	0.66
**100 mg**	**Post-SG (gastric pH=5)**	**Post-OAGB (gastric pH=7)**
C_{max}	6.18	42.88
t_{max}	-20.00	-60.00
AUC_{0-inf}	0.00	1.92

[a] Calculated as: $(value_{pre\text{-}surgery}-value_{post\text{-}surgery}) \times 100/value_{pre\text{-}surgery}$;
